# In Situ Force Microscopy to Investigate Fracture in Stretchable Electronics: Insights on Local Surface Mechanics and Conductivity

**DOI:** 10.1021/acsaelm.2c00328

**Published:** 2022-06-14

**Authors:** Giorgio Cortelli, Luca Patruno, Tobias Cramer, Beatrice Fraboni, Stefano de Miranda

**Affiliations:** † Department of Civil, Chemical, Environmental and Materials Engineering, University of Bologna, Viale del Risorgimento 2, 40136 Bologna, Italy; ‡ Department of Physics and Astronomy, University of Bologna, Viale Berti Pichat 6/2, 40127 Bologna, Italy

**Keywords:** stretchable conductors, in situ AFM, fracture, local conductivity, hard on soft

## Abstract

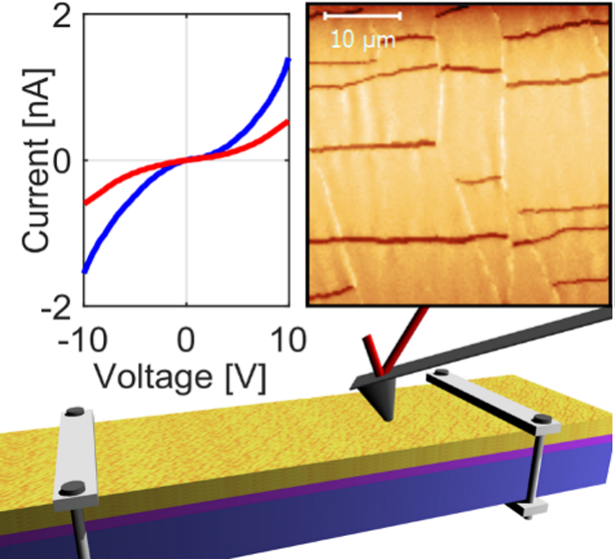

Stretchable conductors are of crucial relevance for emerging technologies such as wearable electronics, low-invasive bioelectronic implants, or soft actuators for robotics. A critical issue for their development regards the understanding of defect formation and fracture of conducting pathways during stress–strain cycles. Here we present a combination of atomic force microscopy (AFM) methods that provides multichannel images of surface morphology, conductivity, and elastic modulus during sample deformation. To develop the method, we investigate in detail the mechanical interactions between the AFM tip and a stretched, free-standing thin film sample. Our findings reveal the conditions to avoid artifacts related to sample bending modes or resonant excitations. As an example, we analyze strain effects in thin gold films deposited on a soft silicone substrate. Our technique allows one to observe the details of microcrack opening during tensile strain and their impact on local current transport and surface mechanics. We find that although the film fractures into separate fragments, at higher strain a current transport is sustained by a tunneling mechanism. The microscopic observation of local defect formation and their correlation to local conductivity will provide insight into the design of more robust and fatigue resistant stretchable conductors.

## 1 Introduction

Stretchable conductive thin films on polymeric substrates are of pivotal importance for several novel applications such as flexible and wearable electronics, stretchable bioelectronic implants, microelectromechanical systems, or soft actuators for robotics.^1−3^ In these applications, the electrical properties of the thin film have to withstand the mechanically demanding deformations occurring during device operation and wear.^4^ A fundamental problem regards the mismatch in elastic properties between conductive thin film and the dielectric substrate material. Conductivity relies on rigid metals or conducting polymers, whereas the substrate is made of soft elastomers to warrant device compliance. Differences in elastic moduli spanning orders of magnitude are often the case and lead to the buildup of interfacial stress during deformation. The consequences are defect formation and defect evolution as observed in the form of thin film necks, cracks, fracture, and delamination. Understanding the microscopic mechanism of defect formation as well as the impact of defects on the electric properties is of paramount importance to optimize the mechanical wear resistance as needed in future application scenarios. Despite this need, microscopy techniques that characterize local morphological, mechanical, and electric properties of the metal layers in situ during the deformation process are still missing.^5,6^ Only such multichannel imaging techniques will ultimately enable the correlation of morphological defects to the electrical response.

To date, several studies demonstrate optical or electron microscopy techniques combined with mechanical stretching to provide rapid imaging of the metallic surface during sample deformation.^1−3,7−9^ Digital image analysis allows one to quantify the local strain field and to obtain quantitative information on the onset of crack formation, the crack length, and the crack density as a function of the strain.^6,10−12^ These are all parameters of central importance to describe the fracture mechanics of such thin films. The drawback of optical or electronic imaging techniques comes from the reflection-based image reconstruction that cannot provide quantitative information on surface height changes. Accordingly, it is difficult to clearly distinguish through and part-through surface cracks or necking structures in tensile strain experiments or to distinguish bulged structures from delaminated ones. Instead, quantitative morphological information is provided by atomic force microscopy (AFM) or confocal laser scanning microscopy. First reports demonstrate the applicability of these microscopy techniques for in situ experiments combined with macroscopic mechanical testing and conductivity measurements.^13^ Such data is highly needed to establish quantitative models for predicting the degradation of conductivity as a function of strain.^1,3,4,12,14−16^

Despite these successes, several crucial local properties of the metal thin film remain experimentally inaccessible, hampering the development of more precise and realistic mechanical models. This regards in particular local conductivity, which is notoriously influenced by the development of the crack pattern. In fact, current models assume ohmic conductance in the defect-free parts of the metal layer, whereas through-thickness cracks are considered as completely isolating barriers.^17,18^ Relying on these two assumptions, the determination of the conductivity of the cracked metallic film reduces to the determination of the geometry of the ohmically conducting pathway connecting through the fractured film. Once it is known, one can estimate the increase in the effective path length and its width reduction during the fracture process to predict the reduction in macroscopic conductivity. So far, no experimental confirmation has been obtained for these central assumptions. Observations such as the degradation of conductivity at large strain values already point to a more complicated role of local conductivity.^17^

To address these issues, we report here an in situ atomic force microscopy method that provides multichannel images of local surface morphology, mechanics, and conductivity on strained metal thin films. The method employs fast repetitive force spectroscopy experiments combined with a conducting AFM probe. Its application on a free-standing strained sample is demonstrated, allowing efficient acquisition of multichannel images at different strain values.

Possible artifacts due to substrate bending or resonant vibration are analyzed in detail to derive the optimized experimental conditions for AFM measurements on free-standing samples. As an example, we investigate the fracture of a thin gold film deposited with an adhesion layer on silicon elastomer substrates. Similar films have important roles as stretchable conductor lines in implantable electronics.^19−22^ The combination of topographic, micromechanical, and electrical imaging channels in our microscopy technique allows one to clearly distinguish different defect types and to understand their effect on the macroscopic properties. For example, we find that although the macroscopic conductivity shows linear ohmic conducting behavior, the local current paths connecting individual fragments behave in a strongly nonlinear way at increasing strain. The observation points to the relevance of tunneling based transport processes in microcracked geometries.

## 2 Experimental Section

### PDMS/Cr/Au Preparation

Polydimethylsiloxane (PDMS) was obtained by mixing cross-linker and Sylgard 184 silicone in a ratio of 1:8. After intensive stirring, the mixture was degassed to remove air bubbles. A layer of a few micrometers of polyacrylic acid (PAA) was spin-coated on the glass substrate before casting PDMS to decrease the adhesion. After pouring the PDMS mixture, it was allowed to set for 20 min to spread homogeneously onto the glass. Samples were then stored for 1 h at 70 °C in an oven. Then the chromium adhesion layer (5 nm thickness) and gold (18 nm thickness) were deposited on the glass/PDMS substrates by thermal evaporation (source sample distance = 25 cm, vacuum pressure = 5.5 × 10^–6^ mbar). Before clamping the sample in the strain stage, the sample was manually bent and twisted to precrack the gold surface to avoid the formation of a single crack cutting all conductive paths. During release from the glass carrier, the sample is subjected to tensile strain as well as bending and twisting. Forces leading to these deformations are applied with a plastic tweezer and are of about 0.5 N. The deformations cause microcrack formation in the metallic coating. The crack pattern is similar to observations described in the literature.^20,22−25^ The electrical contacts were made with copper tape and a conductive epoxy silver-based (without the hardener to keep it liquid) to increase the contact stability during strain variation.

### AFM Probe

A Park System NX10 atomic force microscope was used in the experiments. The Rocky Mountain Nanotechnology probe 25Pt300B was used to perform fast repetitive force spectroscopy. The AFM tip used for resonant frequencies investigation was PPP CONTSCR, while for thickness measurement it was NCHR, with both being from Nanosensors. Before each experiment, the tip sensitivity and force constant are calibrated by an indentation on a silicon surface and thermal tune method. The AFM tips 25Pt300B, PPP CONTSCR, and NCHR have spring constants equal to 18, 0.2, and 5 N m^–1^, respectively.

### AFM Resonant Frequency Investigation

**Figure 1:**
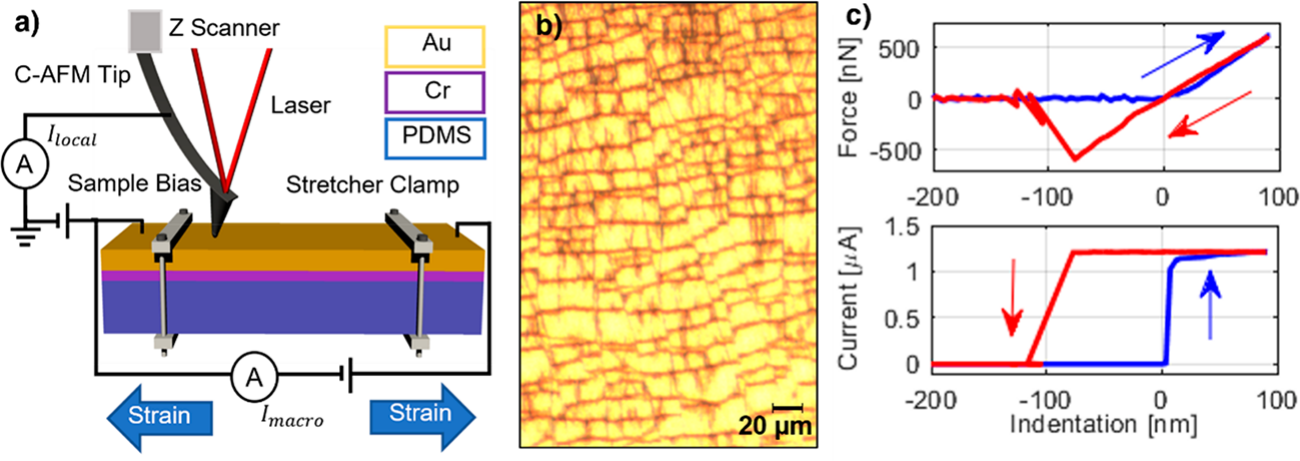
Experimental design of multichannel AFM in situ experiments. (a) Scheme of the experimental setup. The stretcher used to apply tensile strain to the sample is represented by its clamps. The electrical circuit permits application of bias between the tip and sample to measure local currents entering the conducting AFM tip and to measure also the macroscopic sample conductivity. (b) Optical microscopy image of the investigated microcracked gold layer. (c) Force–indentation and current–indentation curves obtained in a single-pixel acquisition. Blue and red arrows represent the load and unload, respectively.

A loudspeaker (Visaton K50) was used as a source of acoustic waves and controlled by a function generator (integrated in the Zurich Instruments MFLI Lock-in Amplifier). The vibrations of the sample were probed by using a very soft AFM tip (PPP CONTSCR) in contact with a set point force of 10 nN. The position sensitive photo detector voltage signal (*V*_a-b_) was recorded by using a lock-in amplifier as a function of the loudspeaker excitation frequency. Since the impact of a 20 nm thick metal layer on the resonance frequencies is considered negligible, we performed the experiments on pure PDMS substrates (2.1 mm thick). The strain stage shown in Figures 1a and S1 was used to apply different tensile strain values, and sample oscillation was measured in the frequency range from 10 Hz to 40 kHz.

### AFM Multichannel Imaging

Height, stiffness, and current maps were generated by performing a fast force spectroscopy for each pixel of the image. Working in contact mode, before fast force spectroscopy, the approach of the AFM tip to the sample surface is performed. The approach is concluded when the set point force is reached. To move from pixel to pixel, the tip is lifted from the substrate. Then the XY stage is moved to place the new pixel below the tip and a tip approach is performed before the next force spectroscopy starts. As force spectroscopy parameters, we set the maximum force to be 500 nN while the speed of the tip along the *Z*-axis was 100 μm s^–1^. We set the lift height as 1 μm, the time for the pixel-to-pixel motion as 5 ms, and a preapproach time of 100 μs. These parameters were optimized on PDMS/Cr/Au surfaces, and acquisition of 128 × 128 pixel images of 40 × 40 μm^2^ sample area was achieved in ca. 30 min. Current maps were obtained by applying a potential difference of 100 mV for strain 0% and 10 V for greater strain values, between the sample and the tip. The macroscopic *I*–*V* curves were acquired with a source meter unit (SMU), tuning the voltage between −7 and 7 V, and measuring the current.

## 3 Results

Our in situ atomic force microscopy technique is based on the experimental setup shown in Figure 1a. This setup provides multichannel acquisitions to map the surface morphology, micromechanics, and local conductivity as a function of the strain applied to the sample. A conductive AFM probe is used to perform simultaneously conductive AFM and force spectroscopy. During a conductive AFM experiment, a bias voltage is applied between the AFM probe and the sample to measure the local electrical current (*I*_local_) that enters the probe through the contact area with the thin film. At the same time, the macroscopic current (*I*_macro_) flowing in the entire sample is measured with a SMU. Uniaxial tensile strain is applied by a custom-designed strain stage in which a screw controls the distance between two clamps that hold the free-standing sample (Figure S1). The dimensions of the sample holder of the strain stage are 2 × 3.5 cm^2^. The multichannel images are obtained through fast repetition force spectroscopy. Each pixel of the image corresponds to force spectroscopy performed with a conductive probe and can thus provide information on surface height, stiffness, and local current measured at a threshold force.

To test the AFM method, we analyzed the strain response of a thin gold film deposited with a chromium adhesion layer on a silicon elastomer substrate. Such films are considered a prototype of a stretchable conductor, as during strain a pattern of microcracks evolves, which absorbs the strain by 3D deformation while maintaining an interconnected conductive pathway in the gold layer.^25^Figure 1b shows an optical microscopy image of such a microcracked film with a gold thickness of 18 nm and PDMS substrate of 2.1 mm thickness as investigated in our experiments. A typical measurement curve acquired with force spectroscopy and conductive AFM on a gold region is shown in Figure 1c.^26^ During the measurement, the AFM probe is pushed into and retracted from the sample at constant speed while the force, the current, and the tip position relative to the surface (indentation) are measured. When the tip contacts the conductive film, an increase of force and a sudden rise in current are recorded. During indentation, the current reaches a saturation value while the force increases linearly. Upon retraction, the force follows the loading curve as expected for an elastic response. Due to adhesion, the surface sticks to the tip when it is displaced above the surface and a negative force is measured while the current remains stable until the contact is lost during snap off.

**Figure 2:**
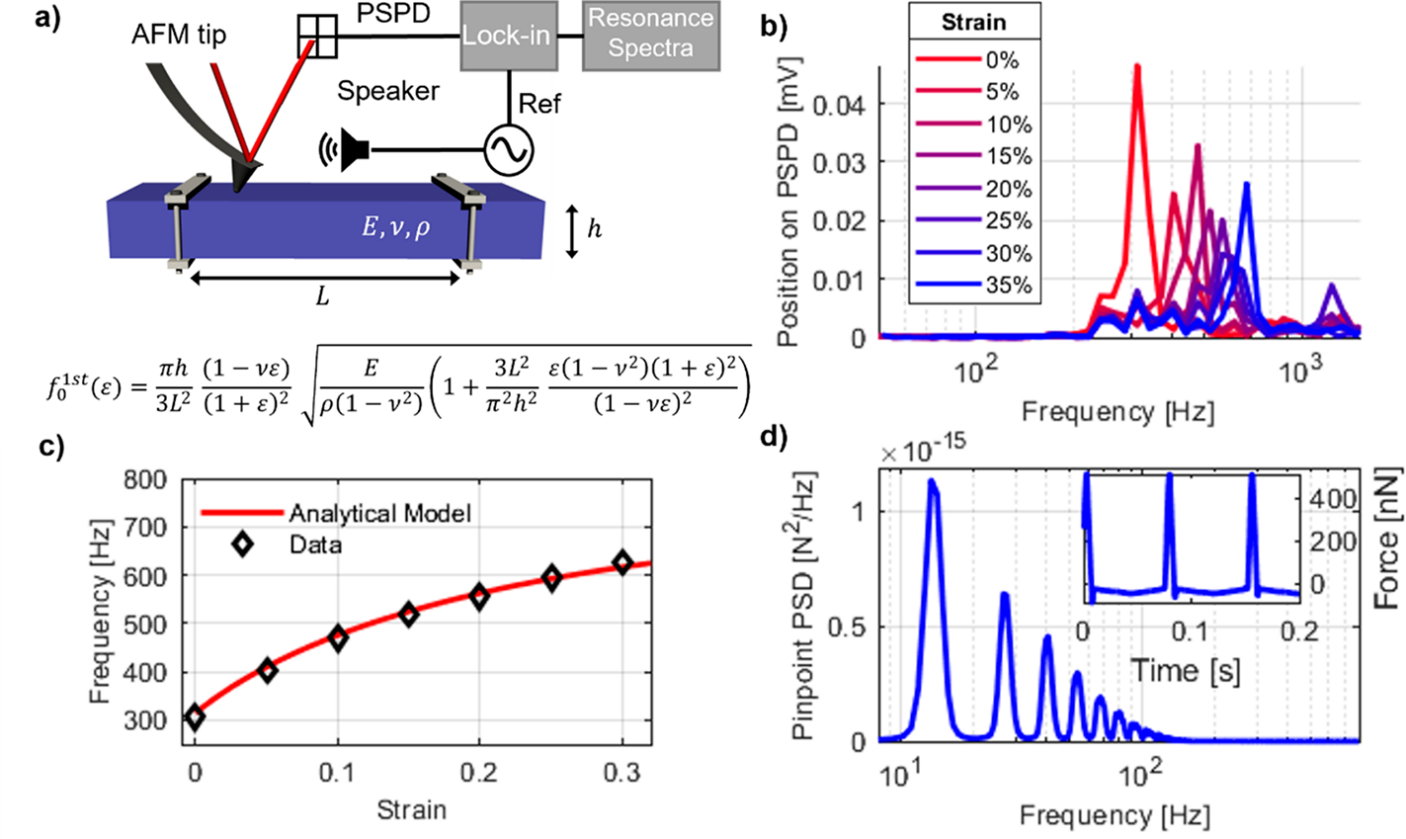
Investigation of measurement artifacts in AFM experiments on free-standing stretched samples. (a) Scheme of the experimental setup to measure the stretched sample’s resonant frequencies. Oscillation modes are excited by sound produced at different frequencies. The response of the sample is measured with AFM in contact mode. (b) Sample oscillation frequency spectra at different strains. Note that the peak corresponding to the first mode moves to the right as the strain increases. (c) Peak frequency, extracted from the data in Figure 2b, as a function of strain. The red line corresponds to the analytical model. (d) Power spectral density of forces between the sample and AFM probe occurring during fast repetitive force spectroscopy acquisition. Note that tip–sample interactions occur at frequencies below the first vibration mode of the free-standing sample, thus excluding possible resonant interactions.

Since the images are obtained through fast repetition of the force spectroscopy experiment, it is crucial to investigate the possible excitation of resonant oscillations of the free-standing sample that would interfere with the AFM characterization. To study resonant oscillations, we used the experimental setup shown in Figure 2a. Oscillation modes were excited by sound produced at different frequencies with a speaker connected to a function generator. The oscillations of the sample were probed with an AFM tip in contact with the sample. The AFM tip deflection signal was recorded with a lock-in amplifier and analyzed as a function of frequency. Figure 2b shows the measured frequency response of the sample vibrations at different strain. As expected, an increase in strain corresponds to a shift of the resonance peak to higher frequencies. To ensure that the AFM measurements are not affected by the sample’s vibrations, the fast repetitive acquisitions must operate at frequencies below the first resonance peak. The resonant frequencies of the sample depend on its dimensions and the strain applied. Therefore, these parameters define limits in which the setup can operate.

To understand the dependence of the resonant frequencies on the sample dimensions and the strain, we developed a model starting from the well-known Rayleigh quotient method for vibration analysis and compared it with the experimental results.^27^ The sample is modeled as a prestressed plate clamped at two ends. Geometrical variations due to large strains and Poisson effects have been accounted for here. In fact, as the strain increases, the length increases while the width and thickness decrease. With these considerations, we obtained the following formula for the first vibrating mode of a rectangular plate clamped at two ends to which a prestress is applied:
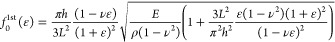
1where *L* and *h* are the length and the thickness of the sample, respectively, while ρ is the density, *E* is the elastic modulus, ν is the Poisson ratio, and ε is the strain. More details can be found in the Supporting Information. We then compared the experimental results with the analytical model. To do so, we considered the dimensions of our sample and the PDMS material parameters (*h* = 2.1 mm, *L* = 19.5 mm, ν = 0.5, ρ = 0.965 g cm^–3^, *E* = 2.21 MPa). The elastic modulus of our PDMS substrate was determined from indentation experiments relying on the Hertz model for the indentation of a rigid spherical tip into an elastic half-space.^28^ As shown in Figure 2c, the analytical model agrees well with the experimental results, even though no fitting parameters are involved. To exclude the excitation of substrate resonant oscillations, the force spectroscopy experiments have to be conducted in a frequency space below the first oscillation peak. Therefore, the repetition rate of the force spectroscopy has to be much smaller than the first mode resonant frequency, as all the next modes will have greater frequencies. In this way, the operation regime is approximately quasi-static and modal amplification is avoided. The power spectral density of tip–sample oscillation modes occurring during the approach and retract movement of the tip is shown in Figure 2d. The inset displays the related time transient of the tip–sample force as caused by vertical movements during consecutive fast force spectroscopy measurements performed in the center of the sample. The sampling rate during the acquisitions was 1.5 kHz, and the total duration was around 80 s. The power spectral density demonstrates that significant frequency components are only below 100 Hz. This is sufficiently lower than the frequency of the first sample oscillation mode starting at 300 Hz to exclude the excitation of sample oscillatory modes and to warrant stable AFM measurement conditions.

A second possible artifact during force spectroscopy measurements on free-standing samples regards global sample deflection. It must be ensured that the displacement due to bending of the free-standing sample, δ_flex_, can be neglected with respect to the local displacement under the tip, δ_hertz_, during indentation. Combining the expressions for beam deflection with the Hertz model, the ratio between the deflection and the indentation is expressed as
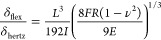
2where is the beam cross section inertia and *B* is the sample width. Considering our experimental case, we obtain δ_flex_/δ_hertz_ = 0.0008. For example, considering 500 nN as the maximum, the deflection of the sample is around 1 nm while the indentation is 1 μm. Therefore, the bending of the sample is negligible with respect to the indentation. For this estimation, we neglected the impact of the thin gold layer. A detailed discussion of eq 2 is provided in the Supporting Information.

**Figure 3:**
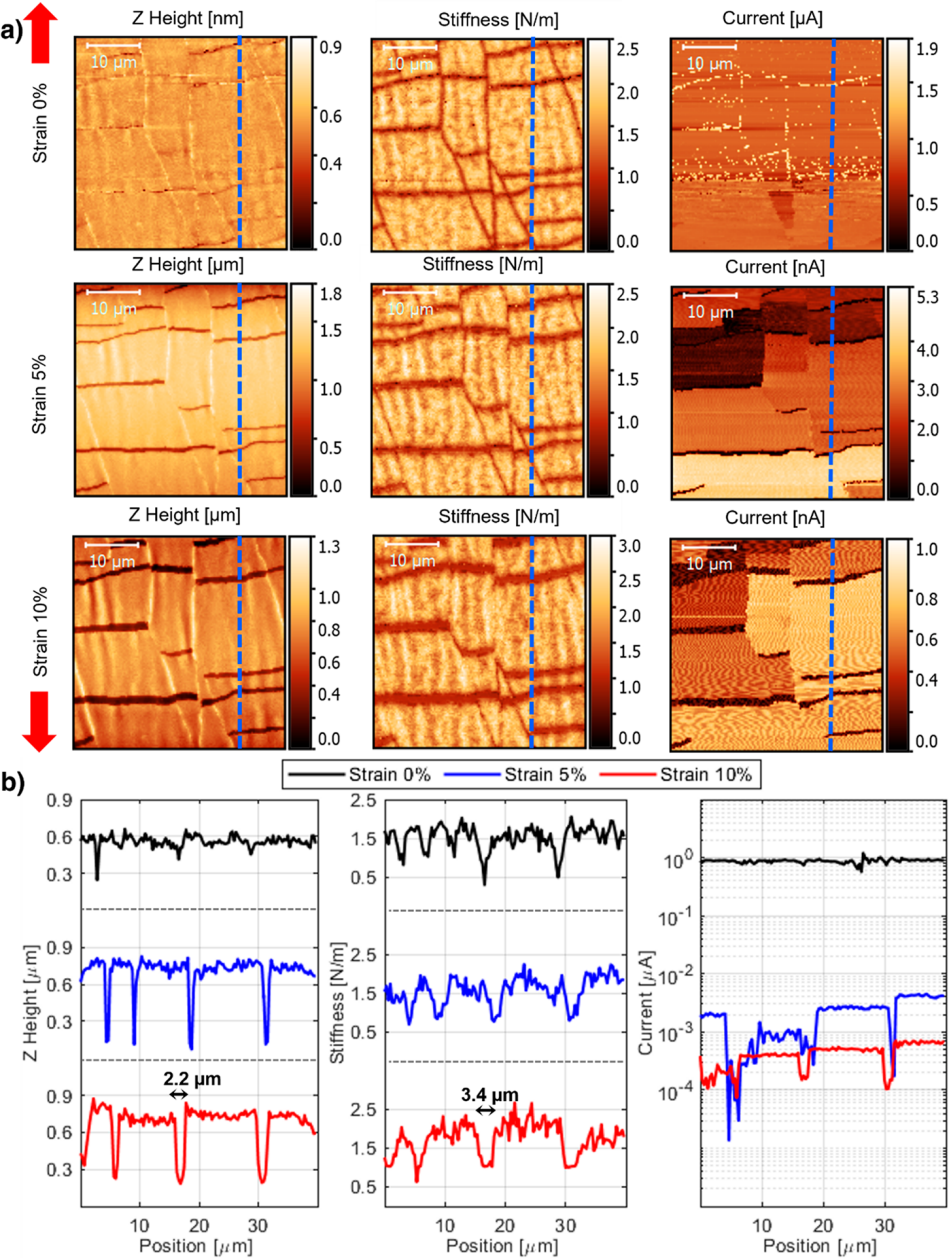
In situ AFM multichannel acquisition. (a) Morphology, stiffness, and current maps as a function of strain acquired on the same region of a microcracked gold film deposited on PDMS elastomer. The strain direction is represented by the two red arrows on the left. (b) Height, stiffness, and current profiles extracted from the AFM maps. The dashed blue lines in (a) indicate the positions of the profiles.

Knowing the operational limits of our setup, we tested the in situ atomic force microscopy method on the stretchable conductor prototype PDMS/Cr/Au. Figure 3 shows the surface height, stiffness, and current maps for three different strain values, 0%, 5%, and 10%, respectively. The blue dashed lines indicate the position of the profile reported at the bottom of Figure 3. The straining direction is indicated by the red arrows. The heights of the morphology map represent the *Z* position when the maximum force value is reached. The stiffness is calculated as the slope of the force–indentation curve, while the local electrical current is measured at maximum force. The morphology maps (*Z* height) and the extracted profiles show that at 0% strain the microcracks are closed. As the strain increases, the size of the microcracks increases. As expected, cracks are oriented in a direction normal to the strain. The maps show that the density of microcracks in the gold thin film is not strain-dependent: no new cracks are formed during the experiment. In the morphology maps at 5% and 10% strain, a second effect is present: the buckling of the gold layer. This is due to the Poisson effect, i.e., the compression of the sample in the direction orthogonal to the applied strain.^29^ In particular, the sample transversal strain is expected to be νε and it is accommodated by the formation of ripples on the sample surfaces, with crests aligned with the applied strain.

The stiffness maps show that the gold film is more compliant near the cracks. In fact, the width of the microcracks in the stiffness maps appears greater than about 1 μm, as measured from the profiles. It can be conjectured that this effect is caused by the loss of rigidity of the gold film when it is indented close to the microcracks.^26^,^30^ Also, note that the stiffness of the gold film does not vary with strain. This indicates that the applied strain has two main effects: widen the microcracks and, through the buckling of the gold layer, create ripples with crests aligned with the straining direction. From the stiffness maps, we estimated the elastic moduli of the metal thin film and the polymeric substrate. Considering the stiffness density distributions shown in Figure a, one can see the presence of two peaks. The lower stiffness peak corresponds to the substrate and is therefore the result of the force spectroscopies performed in the microcracks. As the strain increases, the part of the image where microcracks are present increases. Therefore, the peak is more and more evident. On the other hand, the second peak corresponds to the force spectroscopies made on the regions with the gold film. Fitting the stiffness density distributions with a sum of two Gaussians, we estimated the average stiffness values of the PDMS (dotted lines) and the thin gold film (dashed lines). Given the mean values, we calculated the elastic modulus of PDMS with the Hertz model for a spherical rigid indenter in an infinite half-space, obtaining the following values: *E*_0%_^PDMS^ = 2.8 ± 0.5 MPa, *E*_5%_^PDMS^ = 2.6 ± 0.5 MPa, and *E*_10%_^PDMS^ = 2.8 ± 0.2 MPa. To calculate the elastic modulus of the gold thin film, we used the linear relationship between force and displacement as predicted by the indentation model of rigid thin film deposited on a compliant substrate.^26^ The values obtained are *E*_0%_^Au^ = 92 ± 23 GPa,, *E*_5%_^Au^ = 85 ± 27 GPa, and *E*_10%_^Au^ = 125 ± 43 GPa. The elastic modulus of the gold thin film is found to be comparable with the bulk value.

Current maps shown in Figure 3 were obtained by applying a potential difference of 100 mV for strain 0% and 10 V for greater strain values, between the sample and the tip. At 0% strain, the gold film is entirely conductive, and the presence of the microcracks does not seem to have a major impact. In this regime the measured current is limited by the tip–sample contact. At higher strain values instead, there is a drop in the conductivity of the sample. The measured currents are 3 orders of magnitude lower. Close observation of the current maps and comparison with the height maps allows us to identify the two crucial factors that impact the local current value: First, the height map shows that the microcracks separate the gold thin film into individual fragments, and on the current map we see that each fragment is characterized by a constant current signal. Accordingly, we can conclude that a high conductivity is preserved within a fragment and the current is limited by how the fragment is connected to the rest of the film. Second, the map shows that different fragments are characterized by different current values. This demonstrates that the current is controlled by the transport path that links an individual gold fragment to the device contact. Barriers in the current transport path due to weakly connected fragments insert resistance that reduces the measured current. The current map therefore also contains important information on how the current transport through the microcracked film evolves during strain and highlights the stochastic nature of the fracture process.

**Figure 4:**
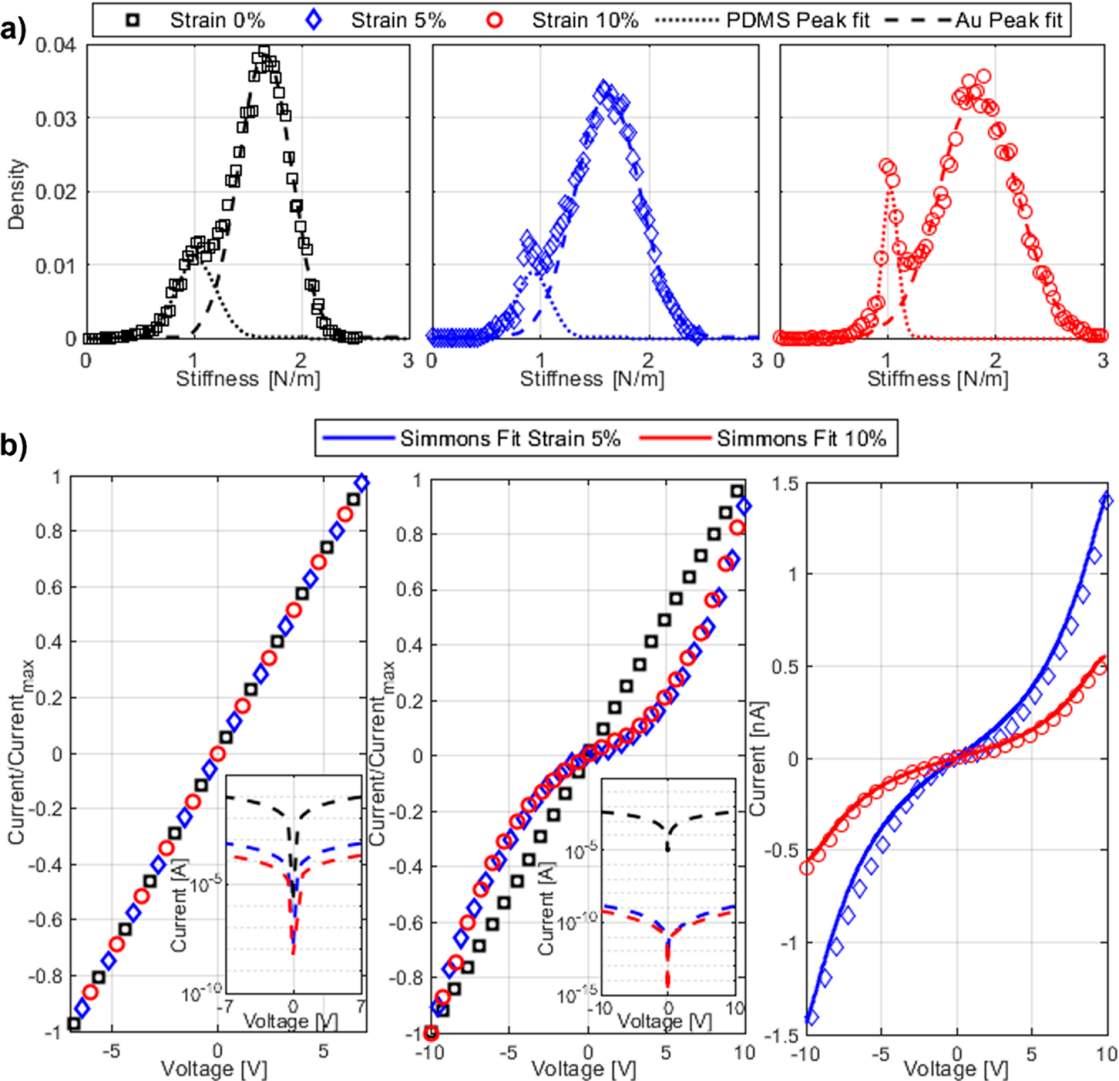
(a) Stiffness histograms obtained from stiffness maps at different strains. The peak at lower stiffness corresponds to the PDMS, while the peak at higher stiffness corresponds to Au thin film. The dotted and dashed lines are Gaussian fits to estimate the average stiffness of PDMS and Au, respectively. (b) The first graph on the left shows the macroscopic *I*–*V* curve of the entire metallic thin film at different strains. The graph in the middle shows the microscopic *I*–*V* curve of a single gold region acquired with C-AFM at different strains. The insets report the same data in a semilogarithmic plot to show the orders of magnitude. The graph on the right shows the microscopic *I*–*V* curve not normalized and fitted with the Simmons model describing the tunneling for a metal–insulator–metal system.

To investigate the current transport onto individual fragments in more detail, we performed conductive AFM *I*–*V* scans at different strain values (Figure 4b). In the figure, we compare the local conducting AFM analysis with the overall current flowing through the sample on a normalized linear scale and logarithmic scale. Both the local AFM current as well as macroscopic sample current show a significant decrease with increasing strain. However, they show different shapes as the strain is increased. The macroscopic *I*–*V* curves maintain a linear, ohmic behavior, while the local current curves show a transition from linear at 0% strain to superlinear at elevated strains. The observed change in shape suggests that charge transport at the microscale between the gold fragments occurs by a field enhanced tunneling effect when strain is applied. Similar measurement curves have been obtained by studying the tunnel effect in gold nanogap junctions.^31,32^ A quantitative description of tunneling transport across metal–insulator–metal systems is provided by the Simmons model.^33^ The model introduces as parameters the insulator width (*s*), the height of the potential barrier (φ), and the overall current scale (*A*), which corresponds to the area of the two metal regions where charge transport by the tunneling effect occurs. The model provides a good fit to our data and the obtained parameter values are reported in Table 1. The barrier height of 5.4 eV and the gap size of a few angstroms indicates a dielectric mediated tunneling mechanism that proceeds through gaps between different gold fragments. Although the significant strain causes a widening of the cracks in the thin film, in the direction orthogonal to the strain, fragments remain closely spaced therefore enabling a tunneling mediated transfer path. In the macroscopic current measurement, the transition to the superlinear behavior is not present because a large number of fragments participate in the transport path. Accordingly, several small cracks have to be overcome and they all induce small potential steps driving field induced tunneling. At individual steps, the potential drop remains well below the tunneling barrier height and a linear response is maintained.

**Table 1:** Parameters of the Simmons Model Estimated by Fitting the Local *I*–*V* Experimental Data

strain	*S* (Å)	φ (eV)	*A* (Å^2^)
5%	2.80 ± 0.04	5.7 ± 0.2	0.29 ± 0.01
10%	2.86 ± 0.02	5.4 ± 0.1	0.11 ± 0.01

## 4 Discussion and Conclusions

Our work demonstrates an in situ experimental method to investigate strain effects in materials and devices for stretchable electronics. Based on fast repetitive force spectroscopy acquisition with a conductive AFM probe, the method enables the microscopic investigation of morphological, mechanical, and electrical properties as a function of strain. The development became possible through a detailed investigation of possible artifacts that can occur when dynamic AFM techniques are performed on a free-standing substrate, only attached at its two ends to the clamps of a tensile stretcher. By deriving and testing the analytical equations that describe substrate deflection and resonant vibrations (eqs 1 and 2), we find the experimental conditions for stable, artifact-free image acquisition. Disturbing sample deformation modes can be reduced by using elastic substrates with a sufficiently large thickness to length ratio and a high elastic modulus or by operating force spectroscopy at slower approach and retract velocities to avoid excitation of resonant modes.

Once the conditions for stable measurements are met, our method provides an unprecedented multichannel imaging technique to correlate morphological defects generated during tensile strain with the mechanical and electrical response. As an example, we analyze the tensile deformation and conductivity changes of a thin gold film deposited with a chromium adhesion layer on a silicon elastomer substrate (PDMS). With our experimental setup, we provide unique insight into the mechanisms of charge transport across gold regions separated by microcracks. For this, the combination of all three imaging channels is crucial: First, the surface height mapping allows us to identify gold fragments surrounded by microcracks and to investigate their morphological evolution during strain. Second, the micromechanical imaging channel provides quantitative measurements of the local elastic modulus. It demonstrates that the externally applied strain is locally absorbed in microcrack widening while gold fragments do not alter their physical extension and stiffness properties as increasing strain. Third, the local conducting properties demonstrate that individual gold fragments remain highly conductive and transport is crucially determined by cracks separating the conducting fragments. We note that, for gold on elastomer films, processing conditions such as gold and adhesion layer thickness as well as pretreatment procedures have a crucial impact on how the microcrack pattern forms and separates islands. In the case studied here, we find at lower strains that a fully conductive, ohmic pathway remains present even though cracks widen in the direction perpendicular to the strain. Instead, at higher strains, our detailed analysis of local *I*–*V* curves demonstrates the transition from the ohmic regime into a tunneling dominated regime, where local barriers have to be overcome by a field enhanced tunneling mechanism. We associate such barriers to microcracks oriented in the direction parallel to the strain. Only in this direction, cracks do not open significantly, thus leaving fragments in close enough proximity to permit tunneling transfer.

In conclusion, our work demonstrates a new in situ experimental approach to investigate mechanical and electrical properties and their correlation at the microscale. It enables one to map properties of stretched samples as a function of strain and to access experimentally the local electrical properties. Both are crucial aspects to understand and predict the properties of stretchable conductors. Based on fast force spectroscopy measurements, the technique is compatible with different materials applied for stretchable electronics comprising hard metallic as well as softer polymeric conductors. We highlight the value of the method by demonstrating the transition from local ohmic transport to field enhanced tunneling at increasing tensile strain in a microcracked gold layer. So far, models of the conductivity of thin films subjected to strain, assume ohmic transport in the defect-free parts of the metal layer, whereas through-thickness cracks are considered as completely isolating barriers.^17,18^ However, our results show that charge transport occurs even if a conducting fragment is completely surrounded by microcracks due to the tunneling effect, introducing a conduction mechanism that has not been accounted for in models.

## Supporting Information Available

Detailed explanation of the analytical model, images of the strain stage, and experimental setup in the laboratory (PDF)
