# Supplementary material for: In Situ Force Microscopy to Investigate Fracture in Stretchable Electronics: Insights on Local Surface Mechanics and Conductivity
Source: ACS Appl Electron Mater. 2022 Jun 14;4(6):2831–8. doi: 10.1021/acsaelm.2c00328 (PMC9245436; doi:10.1021/acsaelm.2c00328)
Supplement: Supplementary file 1 — el2c00328_si_001.pdf [file el2c00328_si_001.pdf]

Supporting Information

In-situ force microscopy to investigate fracture in stretchable electronics: insights on local surface mechanics and conductivity

Giorgio Cortelli, Luca Patruno, Tobias Cramer*, Beatrice Fraboni, Stefano de Miranda*

G. Cortelli, Dr. L. Patruno, Prof. S. de Miranda
Department of Civil, Chemical, Environmental and Materials Engineering
University of Bologna
Viale del Risorgimento 2, 40136, Bologna, Italy

Prof. T. Cramer, Prof. B. Fraboni
Department of Physics and Astronomy
University of Bologna
Viale Berti Pichat 6/2, 40127, Bologna, Italy

E-mail: luca.patruno@unibo.it, tobias.cramer@unibo.it

The multichannel images of the in-situ AFM experimental method proposed in this work are obtained through fast repetition of the force spectroscopy experiment. Therefore, it is crucial to investigate the possible excitation of resonant oscillations of the free-standing sample that would interfere with the AFM acquisitions. A picture of the strain stage is shown in **Figure S1a**, while a picture of the experimental setup showing the free-standing configuration, is reported in **Figure S1b**. To understand the dependence of the resonant frequencies on the sample geometry and the strain, we developed a model starting from the well-known Rayleigh quotient method for vibration analysis and compared it with the experimental results.[25] The sample is modeled as a pre-stressed rectangular plate clamped at two ends. Geometrical variations due to large strains and Poisson effect have been accounted for. In fact, as the strain increases, the length increases, while the width and thickness decrease. Denoting with u the transverse displacement of the plate and with x the axis on the plate mid-surface and orthogonal to the clamps, we assumed $u\,(x) = \frac{1}{2}\left(1 - cos\left(\frac{2\pi x}{L}\right)\right)$ as approximating function for the first

vibration mode of the plate (cylindrical bending), with $x \in [0, L]$. With these assumptions, using the Rayleigh quotient method, we obtained the following formula for the frequency of the first vibration mode:

$$f_0^{1st}(\varepsilon) = \frac{\pi h}{3L^2}\frac{(1-\nu\varepsilon)}{(1+\varepsilon)^2}\sqrt{\frac{E}{\rho(1-\nu^2)}\left(1+\frac{3L^2}{\pi^2 h^2}\frac{\varepsilon(1-\nu^2)(1+\varepsilon)^2}{(1-\nu\varepsilon)^2}\right)} \tag{S1}$$

where L and h are the length and the thickness of the sample, respectively, while ρ is the density, E is the elastic modulus, ν is the Poisson's ratio and ε is the strain.

We then investigated the impact on force-indentation curves of the bending of the sample. In fact, considering the force applied from the AFM tip as a concentrated force, it must be ensured that the bending of the free-standing sample can be neglected with respect to the indentation of the tip inside the sample. To get an estimate of the bending at the center of the sample, we assumed the plate to behave like a beam clamped on both ends. The stiffness of the beam is then given by the formula:

$$K_{flex} = \frac{192EI}{L^3(1-\nu^2)} \tag{S2}$$

where $I = \frac{Bh^3}{12}$ is the beam cross section inertia, and B is the sample width. Notice that such approach is valid only if B is smaller than L, and the error for $B = L$ is approximately 10 %. The plate deflection is then $\delta_{flex} = F/K_{flex}$. The validity of this description has been confirmed with numerical simulations. An estimate of the indentation of the AFM tip on the PDMS sample is given by the well-known Hertz model for a rigid spherical indenter in an infinite half-space. Therefore, it can be expressed as $\delta_{hertz} = \left(\frac{3F(1-\nu^2)}{4E\sqrt{R}}\right)^{2/3}$. The ratio of δ_{flex} and δ_{hertz} allows understanding of which between indentation and bending is the dominant term. The ratio can be expressed as:

$$\frac{\delta_{flex}}{\delta_{hertz}} = \frac{L^3}{192I}\left(\frac{8FR(1-\nu^2)}{9E}\right)^{1/3} \tag{S3}$$

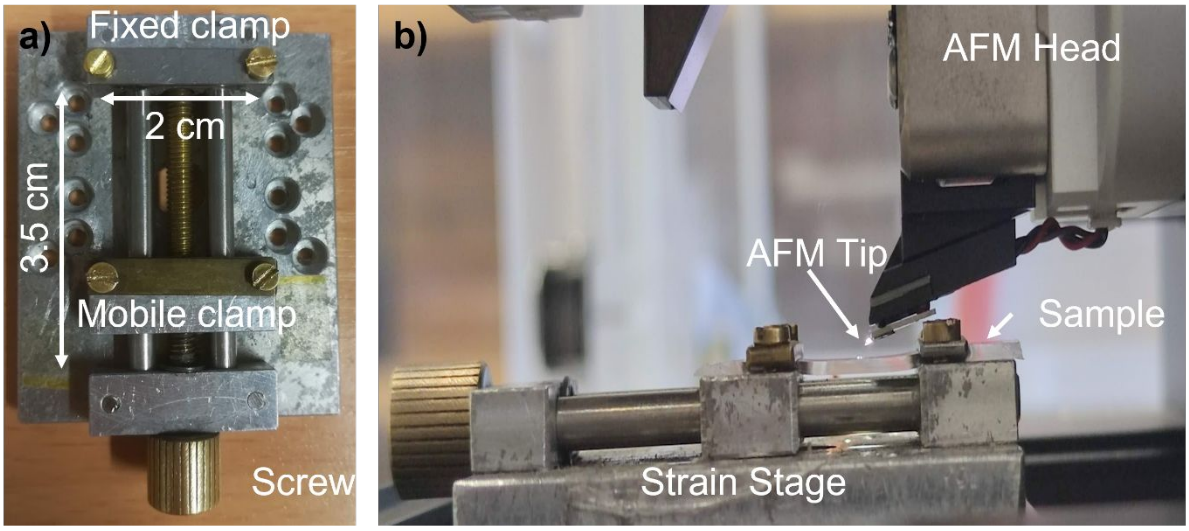

Figure S1. a) Photo of the strain stage with its dimensions. b) Photo of the experimental setup with sample clamped to the strain stage under the AFM tip.

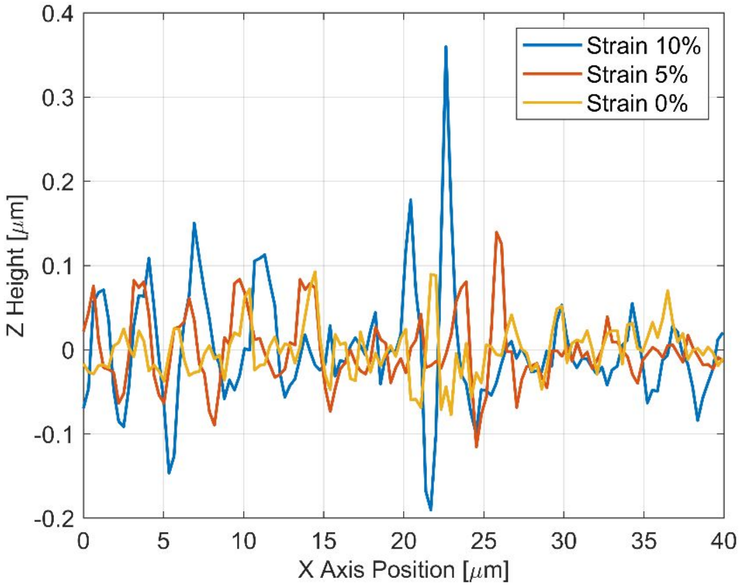

Figure S2. Cross-section of the sample morphology extracted orthogonally to the direction of tensile stress (i.e., the horizontal profiles of **Figure 3** in the main manuscript). The profiles show the buckling due to the Poisson effect at strains greater than 0%.
